# Supplementary material for: Molecular Basis of CO2 Sensing in Hyphantria cunea
Source: Int J Mol Sci. 2024 May 30;25(11):5987. doi: 10.3390/ijms25115987 (PMC11172650; doi:10.3390/ijms25115987)
Supplement: Supplementary file 1 [file ijms-25-05987-s001.zip › Figures S1-S3.pdf]

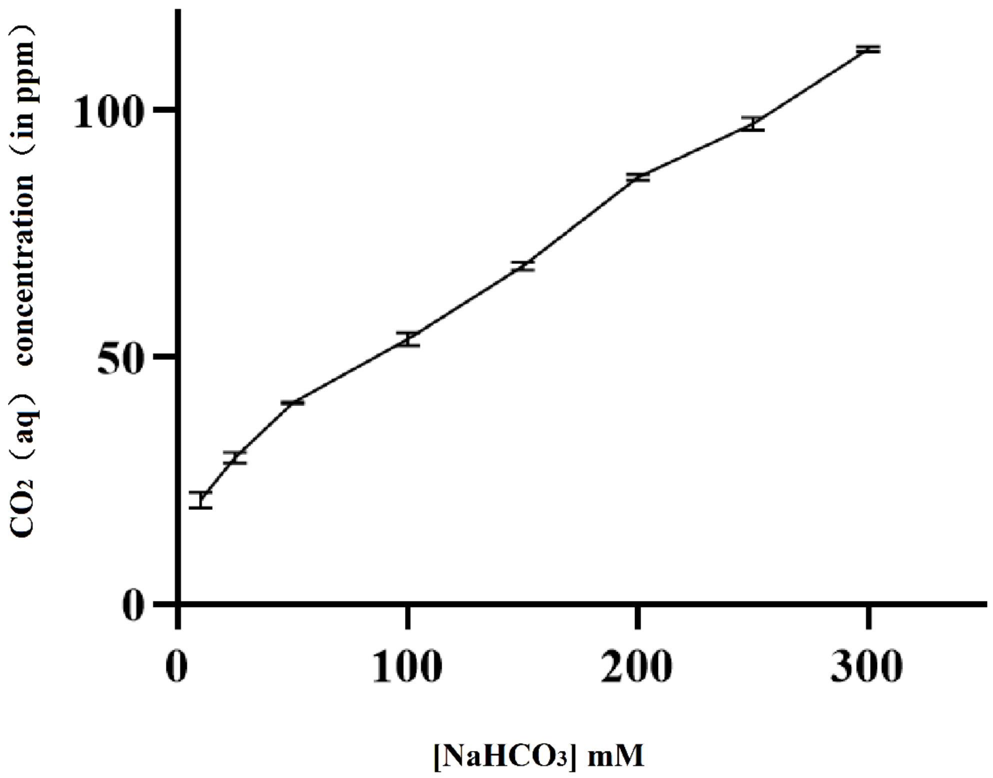

Figure S1. Concentration of CO_2 dissolved in different concentrations of $NaHCO_3$ solution.

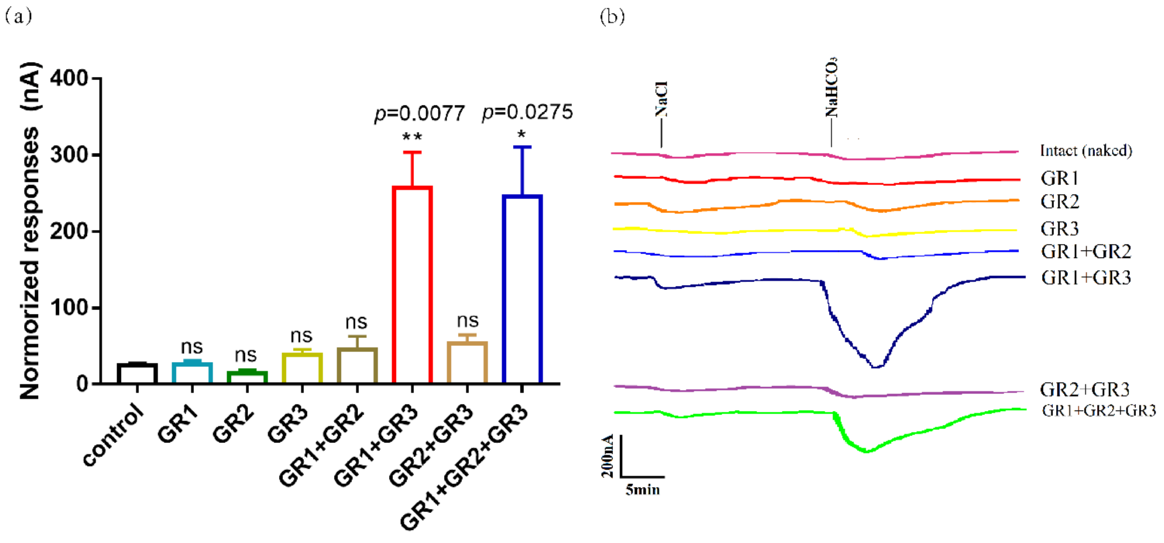

Figure S2. TEVC response of *HcunGR1, HcunGR2, HcunGR3* alone and their combinations to 100Mm of NaCl and $NaHCO_3$. (a) Concentration response histogram of *HcunGR1*, *HcunGR2*, *HcunGR3* alone and their combinations to $NaHCO_3$; (b) Response of non-injected; (c) Response of *HcunGR1*; (d) Response of *HcunGR2*; (e) Response of *HcunGR3*; (f) Response of *HcunGR1+HcunGR2*; (g) Response of *HcunGR1+HcunGR3*; (h) Response of *HcunGR2+HcunGR3*; (i) Response of

HcunGR1+HcunGR2+HcunGR3. N = 3 biological replicates; The data are presented as the mean ± SEM. Statistical differences were evaluated by unpaired t tests. *$p < 0.05$, **$p < 0.01$.

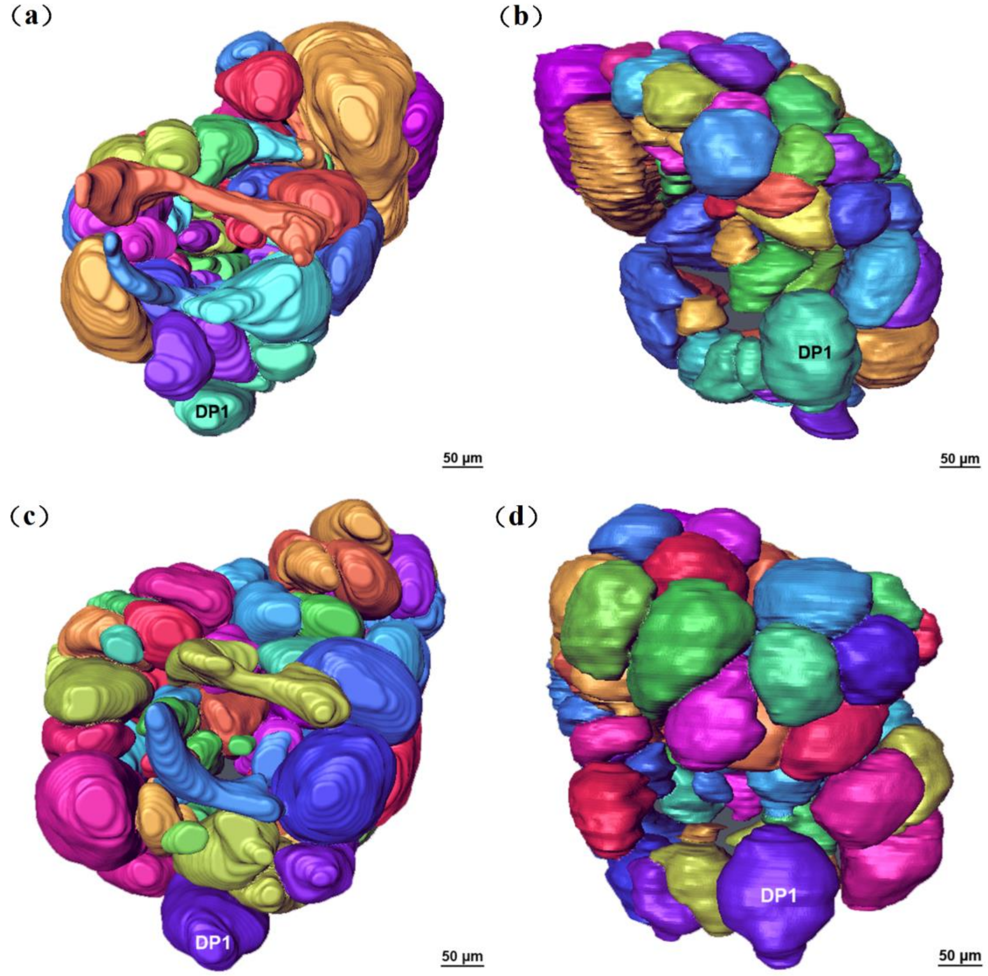

Figure S3. Three-dimensional structure of the antennal lobe of *H. cunea* observed from two angles. (a) Image of male *H. cunea* from dorsal view; (b) Image of male *H. cunea* from posterior view; (c) Image of female *H. cunea* from dorsal view; (d) Image of female *H. cunea* from posterior view. Scale bar = 50 μm.
