# Supplementary material for: Molecular Basis of CO2 Sensing in Hyphantria cunea
Source: Int J Mol Sci. 2024 May 30;25(11):5987. doi: 10.3390/ijms25115987 (PMC11172650; doi:10.3390/ijms25115987)
Supplement: Supplementary file 1 [file ijms-25-05987-s001.zip › Tables S1-S6.pdf]

Table S1 Detailed information on identified *HcunGRs*.

Gene	Full-length	ORF (aa)	BLASTX best hit				
			Description	Query cover	E-value	Identity	Accession
HcunGR1	Yes	467	gustatory and odorant receptor 22 [Helicoverpa armigera]	97	0.0	88.65	EVM0013299
HcunGR2	Yes	434	gustatory and odorant receptor 22-like [Trichoplusia ni]	99	0.0	91.44	EVM0008460
HcunGR3	Yes	479	gustatory receptor 2 [Peridroma saucia]	100	0.0	85.80	EVM0000467
HcunGR4	Yes	402	gustatory receptor 8 [Peridroma saucia]	98	3e-167	59.60	EVM0007040
HcunGR5	Yes	317	gustatory receptor for sugar taste 64f [Helicoverpa armigera]	91	1e-81	41.75	EVM0009821
HcunGR6	No	114	gustatory receptor for sugar taste 64f-like [Vanessa atalanta]	86	3e-31	59.60	EVM0004185
HcunGR7	No	183	antennal gustatory receptor 2 [Dendrolimus punctatus]]	93	3e-48	48.84	EVM0014036
HcunGR8	No	354	gustatory protein 6 [Spodoptera frugiperda]	93	6e-137	61.88	EVM0001407
HcunGR9	No	322	gustatory receptor 6 [Spodoptera litura]	87	4e-124	59.76	EVM0010391

Gene	Full-length	ORF (aa)	BLASTX best hit				
			Description	Query cover	E-value	Identity	Accession
HcunGR10	Yes	324	gustatory receptor 5a for trehalose-like [Helicoverpa]	89	1e-154	69.69	EVM0010760
HcunGR11	Yes	440	gustatory receptor for sugar taste 43a [Helicoverpa armigera]	92	1e-43	30.42	EVM0002678
HcunGR12	Yes	386	gustatory receptor 5a for trehalose-like [Spodoptera litura]	99	7e-171	59.26	EVM0007620
HcunGR13	Yes	253	gustatory receptor for sugar taste 64f-like [Achroia grisella]	98	2e-61	35.00	EVM0008792
HcunGR14	No	226	gustatory receptor 11 [Spodoptera litura]	82	3e-73	56.87	EVM0001524
HcunGR15	No	233	TPA: gustatory receptor 26 [Bombyx mori]	86	6e-18	27.36	EVM0011900
HcunGR16	Yes	490	gustatory receptor 4 [Helicoverpa armigera]	94	0	83.62	EVM0009444
HcunGR17	No	107	gustatory receptor 24 [Achelura yunnanensis]	83	6e-08	30.34	EVM0014514
HcunGR18	No	202	gustatory receptor 48 [Papilio machaon]	95	2e-65	48.18	EVM0009134
HcunGR19	Yes	351	gustatory receptor for sugar taste 43a [Helicoverpa armigera]	91	2e-37	32.11	EVM0003031

Gene	Full-length	ORF (aa)	BLASTX best hit				
			Description	Query cover	E-value	Identity	Accession
HcunGR20	Yes	437	gustatory receptor 65 [Spodoptera litura]	93	2e-110	42.17	EVM0001187
HcunGR21	Yes	443	gustatory receptor 65 [Spodoptera litura]	93	1e-107	43.63	EVM0014257
HcunGR22	No	255	gustatory receptor 53 [Operophtera brumata]	63	2e-34	44.17	EVM0007983
HcunGR23	Yes	243	gustatory receptor 51 [Bombyx mori]	94	3e-78	56.47	EVM0005122
HcunGR24	Yes	392	gustatory receptor 45 [Bombyx mori]	99	1e-81	37.87	EVM0005410
HcunGR25	Yes	273	gustatory receptor 202 [Spodoptera litura]	70	7e-29	37.5	EVM0000959
HcunGR26	Yes	286	gustatory receptor 202 [Spodoptera litura]	69	8e-24	33.01	EVM0012398
HcunGR27	Yes	300	gustatory receptor 227 [Spodoptera litura]	75	6e-33	34.93	EVM0011573
HcunGR28	No	129	gustatory receptor 227 [Spodoptera litura]	100	6e-19	36.36	EVM0013318
HcunGR29	Yes	283	gustatory receptor 99 [Spodoptera litura]	98	3e-25	28.72	EVM0013404

Gene	Full-length	ORF (aa)	BLASTX best hit				
			Description	Query cover	E-value	Identity	Accession
HcunGR30	Yes	250	hypothetical protein HW555_013559 [Spodoptera exigua]	98	5e-39	39.37	EVM0009415
HcunGR31	No	164	gustatory receptor 14 [Heortia vitessoides]	99	1e-06	26.55	EVM0014580
HcunGR32	No	177	gustatory receptor 227 [Spodoptera litura]	85	5e-24	37.74	EVM0004939
HcunGR33	No	199	gustatory receptor 27 [Achelura yunnanensis]	98	9e-22	30.50	EVM0009320
HcunGR34	No	188	gustatory receptor 227 [Spodoptera litura]	95	1e-24	37.02	EVM0008579
HcunGR35	No	186	hypothetical protein SFRURICE_017102 [Spodoptera frugiperda]	98	6e-34	40.44	EVM0006052
HcunGR36	No	193	gustatory receptor 227 [Spodoptera litura]	86	2e-25	39.29	EVM0013759
HcunGR37	No	129	gustatory receptor 99 [Spodoptera litura]	74	7e-16	44.33	EVM0007986
HcunGR38	No	228	gustatory receptor 27 [Achelura yunnanensis]	97	3e-20	27.56	EVM0005904
HcunGR39	Yes	274	gustatory receptor 227 [Spodoptera litura]	72	2e-35	38.42	EVM0008948

Gene	Full-length	ORF (aa)	BLASTX best hit				
			Description	Query cover	E-value	Identity	Accession
HcunGR40	No	113	gustatory receptor 27 [Achelura yunnanensis]	86	5e-16	36.73	EVM0003193
HcunGR41	Yes	273	gustatory receptor 43 [Spodoptera litura]	93	4e-17	23.45	EVM0011675
HcunGR42	No	151	ustatory receptor 68a [Helicoverpa zea]	98	2e-16	32.05	EVM0001267
HcunGR43	Yes	432	gustatory receptor 43 [Spodoptera litura]	96	7e-146	50.12	EVM0003074
HcunGR44	Yes	390	gustatory receptor 68a [Helicoverpa zea]	95	7e-127	57.49	EVM0009024

Table S2 Primers for RT-qPCR of 3 candidate *HcunGR* in *H. cunea.*

Gene	Forward primer (5' to 3')	Reverse primer (5' to 3')
β-actin	TGCTATGTCGCTCTTGACTTCG	CGTTCGTTTCCAATGGTGATGA
HcunGR1	GCCTCTTTGTCATTGTCGGA	ATGGGCGGATTCTTAGCG
HcunGR2	TAGCCGTCATGCCCATAACA	CGTTCATAACCCACCACCAGA
HcunGR3	TCTACGCACCGCAGAAGG	CTCAAAATCAACCCAGCCA

Table S3 Primers for RT-PCR of 3 candidate *HcunGR* in *H. cunea.*

Gene	Forward primer (5' to 3')	Reverse primer (5' to 3')
HcunGR1	GGGTGGGTTATGATTG	ACCGGCTGACTCTGTA
HcunGR2	AGCGACATCGTCATTT	AATCTTCTTCGGGTCA
HcunGR3	ACGCCCTTGATTACTT	CGAATTGTTACGCTGA

Table S4 Raw data for the Electrolabialpalpography (ELPG) concentration-dependence curves of CO_2.

	Relative ELPG response					
CO_2 Concentration	Female			Male		
1%	5.1	5.777777778	6.057142857	3.25	3.35	3.414285714
3%	9.714285714	7	7.214285714	4.4	3.675	3.114285714
5%	8.314285714	10.91111111	9.385714286	5.225	6.925	4.671428571
8%	12.82857143	14.08888889	11.73333333	5.725	6.4	6.357142857
10%	14.02857143	13.525	10.67142857	6.4	6.075	7.371428571

Table S5 Raw data for the Electroantennogram (EAG) concentration-dependence curves of CO_2

	Relative EAG response					
CO_2 Concentration	Female			Male		
1%	1.47826087	1.390243902	1.346666667	1.092307692	1.540384615	1.481761006
3%	2.173913043	1.512195122	1.693333333	1.415384615	2.134615385	1.314049587
5%	2.826086957	2.170731707	1.8	2.184615385	2.5	1.7
8%	3.347826087	2.536585366	2.04	2.753846154	2.721153846	2.3
10%	3.47826087	3.487804878	2.2	3.076923077	2.932692308	3.2

Table S6 Raw data for the Two-electrode voltage clamp recording (TEVC) responses of *HcunGR1*, *HcunGR2*, and *HcunGR3* alone and in combination to different concentrations of NaCl and $NaHCO_3$.

	Normalized responses (nA)							
**Concentration(mM)**	**10**	**25**	**50**	**100**	**150**	**200**	**250**	**300**
	10	25	30	330	403	770	1225	1025
**GR1+GR3**	9	30	70	271	640	760	1388	790
	2	3	25	170	520	780	1550	720
	17	65	50	135	380	410	600	380
**GR1+GR2+GR3**	12	30	40	242	374	500	640	215
	7	25	49	360	416	432	486	375
	5	25	10	35	40	15	106	100
**GR2+GR3**	5	10	30	74	110	188	170	50
	5	10	20	52	5	22	100	120
	2	3	5	23	15	0	40	41
**GR1+GR2**	5	15	40	80	130	110	90	45
	3	20	27	33	80	11	41	150
	6	2	12	17	5	8	70	130
**GR1**	5	14	12	30	30	20	50	40
	6	25	40	32	0	50	30	70
	0	3	8	5	55	40	130	180
**GR2**	6	5	8	15	70	84	60	50
	0	0	30	22	23	135	60	80
	3	0	25	37	10	30	30	30
**GR3**	2	3	3	50	50	40	50	50
	2	1	4	50	95	100	100	30
